# Obstetric Outcomes of SARS-CoV-2 Infection in Asymptomatic Pregnant Women

**DOI:** 10.3390/v13010112

**Published:** 2021-01-15

**Authors:** Monica Cruz-Lemini, Elena Ferriols Perez, Maria Luisa de la Cruz Conty, Africa Caño Aguilar, Maria Begoña Encinas Pardilla, Pilar Prats Rodríguez, Marta Muner Hernando, Laura Forcen Acebal, Pilar Pintado Recarte, Maria del Carmen Medina Mallen, Noelia Perez Perez, Judit Canet Rodriguez, Ana Villalba Yarza, Olga Nieto Velasco, Pablo Guillermo del Barrio Fernandez, Carmen Maria Orizales Lago, Beatriz Marcos Puig, Begoña Muñoz Abellana, Laura Fuentes Ricoy, Agueda Rodriguez Vicente, Maria Jesus Janeiro Freire, Macarena Alferez Alvarez-Mallo, Cristina Casanova Pedraz, Onofre Alomar Mateu, Cristina Lesmes Heredia, Juan Carlos Wizner de Alva, Alma Posadas San Juan, Montserrat Macia Badia, Cristina Alvarez Colomo, Antonio Sanchez Muñoz, Laia Pratcorona Alicart, Ruben Alonso Saiz, Monica Lopez Rodriguez, Maria Carmen Barbancho Lopez, Marta Ruth Meca Casbas, Oscar Vaquerizo Ruiz, Eva Moran Antolin, Maria Jose Nuñez Valera, Camino Fernandez Fernandez, Albert Tubau Navarra, Alejandra Maria Cano Garcia, Susana Soldevilla Perez, Irene Gattaca Abasolo, Jose Adanez Garcia, Alberto Puertas Prieto, Rosa Ostos Serna, Maria del Pilar Guadix Martin, Monica Catalina Coello, Silvia Espuelas Malon, Jose Antonio Sainz Bueno, Maria Reyes Granell Escobar, Sara Cruz Melguizo, Oscar Martinez Perez, on behalf of the Spanish Obstetric Emergency Group

**Affiliations:** 1 Department of Gynecology and Obstetrics, Women and Perinatal Health Research Group, Biomedical Research Institute (IIB-Sant Pau), Santa Creu i Sant Pau University Hospital, 08025 Barcelona, Spain; cruzlemini@gmail.com (M.C.-L.); mmedinam@santpau.cat (M.d.C.M.M.); 2 Department of Gynecology and Obstetrics, Parc de Salut Mar University Hospital, 08003 Barcelona, Spain; eferriols@parcdesalutmar.cat (E.F.P.); sespuelas@parcdesalutmar.cat (S.E.M.); 3 Fundacion de Investigacion Biomedica, Puerta de Hierro University Hospital of Majadahonda, 28222 Madrid, Spain; farmcruz@gmail.com; 4 Department of Gynecology and Obstetrics, San Cecilio University Hospital of Granada, 18016 Granada, Spain; africacano59@gmail.com; 5 Department of Gynecology and Obstetrics, Puerta de Hierro University Hospital of Majadahonda, 28222 Madrid, Spain; beenpar@yahoo.es (M.B.E.P.); saracruz.gine@yahoo.es (S.C.M.); 6 Department of Gynecology and Obstetrics, Dexeus University Hospital, 08028 Barcelona, Spain; pilpra@dexeus.com; 7 Department of Gynecology and Obstetrics, La Paz University Hospital, 28046 Madrid, Spain; mlm_marta@hotmail.com; 8 Department of Gynecology and Obstetrics, Doce de Octubre University Hospital, 28041 Madrid, Spain; lauratrona@gmail.com; 9 Department of Gynecology and Obstetrics, Gregorio Marañon University Hospital, 28007 Madrid, Spain; ppintadorec@yahoo.es; 10 Department of Gynecology and Obstetrics, San Carlos University Hospital, 28040 Madrid, Spain; perezpereznoelia@yahoo.es; 11 Department of Gynecology and Obstetrics, Santa Caterina Hospital, 17190 Girona, Spain; judit.canet@ias.cat; 12 Department of Gynecology and Obstetrics, University Hospital of Salamanca, 37007 Salamanca, Spain; avillalba@saludcastillayleon.es; 13 Department of Gynecology and Obstetrics, Quironsalud Madrid University Hospital, 28223 Madrid, Spain; onietovela@hotmail.com; 14 Department of Gynecology and Obstetrics, Getafe University Hospital, 28905 Madrid, Spain; pablobarri@gmail.com; 15 Department of Gynecology and Obstetrics, Severo Ochoa University Hospital, 28911 Madrid, Spain; carmen.orizales@salud.madrid.org; 16 Department of Gynecology and Obstetrics, La Fe University and Polytechnic Hospital, 46026 Valencia, Spain; beatrizmarcospuig@gmail.com; 17 Department of Gynecology and Obstetrics, Sant Joan de Reus University Hospital, 43204 Tarragona, Spain; begomunoz@hotmail.com; 18 Department of Gynecology and Obstetrics, University Hospital of Ferrol, 15405 A Coruña, Spain; laura.fuentes.ricoy@sergas.es; 19 Department of Gynecology and Obstetrics, Doctor Josep Trueta University Hospital of Girona, 17007 Girona, Spain; aguedarv@gmail.com; 20 Department of Gynecology and Obstetrics, University Hospital Complex of A Coruña, 15006 A Coruña, Spain; mjaneirof@yahoo.es; 21 Department of Gynecology and Obstetrics, HM Hospitals, 28015 Madrid, Spain; macarena.alferez.am@gmail.com; 22 Department of Gynecology and Obstetrics, University Hospital of Torrejon, 28850 Madrid, Spain; casanovapedraz@hotmail.com; 23 Department of Gynecology and Obstetrics, Regional Hospital of Inca, 07300 Illes Balears, Spain; alomarmateu@gmail.com; 24 Department of Gynecology and Obstetrics, Parc Taulí Hospital, 08208 Barcelona, Spain; clesmes@tauli.cat; 25 Department of Gynecology and Obstetrics, San Pedro de Alcántara Hospital, 10003 Cáceres, Spain; jcwizner@gmail.com; 26 Department of Gynecology and Obstetrics, Rio Hortega University Hospital, 47012 Valladolid, Spain; almapsj@gmail.com; 27 Department of Gynecology and Obstetrics, Arnau de Vilanova University Hospital, 25198 Lleida, Spain; mmaciabadia@gmail.com; 28 Department of Gynecology and Obstetrics, Valladolid University Clinical Hospital, 47003 Valladolid, Spain; calvarezc.cac@gmail.com; 29 Department of Gynecology and Obstetrics, General University Hospital of Ciudad Real, 13005 Ciudad Real, Spain; asanchezm@sescam.jccm.es; 30 Department of Gynecology and Obstetrics, Germans Trias i Pujol University Hospital, 08916 Barcelona, Spain; lpratcorona.mn.ics@gencat.cat; 31 Department of Gynecology and Obstetrics, University Hospital of Burgos, 09006 Burgos, Spain; alonsorub@yahoo.es; 32 Department of Gynecology and Obstetrics, Joan XXIII University Hospital of Tarragona, 43005 Tarragona, Spain; monicalopez4099@gmail.com; 33 Department of Gynecology and Obstetrics, Infanta Sofia University Hospital, 28703 Madrid, Spain; mbarbancholopez@gmail.com; 34 Department of Gynecology and Obstetrics, Poniente Hospital of Almeria, 04700 Almeria, Spain; martameca@hotmail.com; 35 Department of Gynecology and Obstetrics, University Hospital of Cabueñes, 33394 Asturias, Spain; oscar.vaquerizo@sespa.es; 36 Department of Gynecology and Obstetrics, Son Espases University Hospital, 07120 Illes Balears, Spain; emoranantolin@yahoo.es; 37 Department of Gynecology and Obstetrics, Virgen de la Luz Hospital, 16002 Cuenca, Spain; m.jose.nunez.valera@gmail.com; 38 Department of Gynecology and Obstetrics, University Assistance Complex of Leon, 24001 Leon, Spain; caminoffernandez@gmail.com; 39 Department of Gynecology and Obstetrics, Son Llatzer University Hospital, 07198 Illes Balears, Spain; atubau68@icloud.com; 40 Department of Gynecology and Obstetrics, University Hospital of El Tajo, 28300 Madrid, Spain; sandracano80@hotmail.com; 41 Department of Gynecology and Obstetrics, University Hospital of Jerez de la Frontera, 11407 Cadiz, Spain; soldevillasusana@hotmail.com; 42 Department of Gynecology and Obstetrics, Txagorritxu University Hospital of Araba, 01009 Araba, Spain; igastaca@gmail.com; 43 Department of Gynecology and Obstetrics, Central University Hospital of Asturias, 33011 Asturias, Spain; adanezjose@gmail.com; 44 Department of Gynecology and Obstetrics, Virgen de las Nieves University Hospital, 18014 Granada, Spain; apuertas51@hotmail.com; 45 Department of Gynecology and Obstetrics, Virgen de Valme University Hospital, 41014 Sevilla, Spain; rosam.ostos.sspa@juntadeandalucia.es; 46 Department of Gynecology and Obstetrics, Virgen Macarena University Hospital, 41009 Sevilla, Spain; pilarguadix@gmail.com; 47 Department of Gynecology and Obstetrics, Virgen de la Concha Hospital, 49022 Zamora, Spain; mccoello@gmail.com; 48 Department of Gynecology and Obstetrics, G. Chacon (Viamed Santa Angela de la Cruz Hospital), 41014 Sevilla, Spain; jsainz@us.es; 49 Department of Gynecology and Obstetrics, Juan Ramon Jimenez University Hospital, 21005 Huelva, Spain; mrgranell@gmail.com

**Keywords:** SARS-CoV-2, pregnancy, coronavirus, asymptomatic infection, perinatal outcomes, delivery, maternal complications

## Abstract

Around two percent of asymptomatic women in labor test positive for severe acute respiratory syndrome coronavirus 2 (SARS-CoV-2) in Spain. Families and care providers face childbirth with uncertainty. We determined if SARS-CoV-2 infection at delivery among asymptomatic mothers had different obstetric outcomes compared to negative patients. This was a multicenter prospective study based on universal antenatal screening for SARS-CoV-2 infection. A total of 42 hospitals tested women admitted for delivery using polymerase chain reaction, from March to May 2020. We included positive mothers and a sample of negative mothers asymptomatic throughout the antenatal period, with 6-week postpartum follow-up. Association between SARS-CoV-2 and obstetric outcomes was evaluated by multivariate logistic regression analyses. In total, 174 asymptomatic SARS-CoV-2 positive pregnancies were compared with 430 asymptomatic negative pregnancies. No differences were observed between both groups in key maternal and neonatal outcomes at delivery and follow-up, with the exception of prelabor rupture of membranes at term (adjusted odds ratio 1.88, 95% confidence interval 1.13–3.11; *p* = 0.015). Asymptomatic SARS-CoV-2 positive mothers have higher odds of prelabor rupture of membranes at term, without an increase in perinatal complications, compared to negative mothers. Pregnant women testing positive for SARS-CoV-2 at admission for delivery should be reassured by their healthcare workers in the absence of symptoms.

## 1. Introduction

To date, more than 11 million cases of the new severe acute respiratory syndrome coronavirus 2 (SARS-CoV-2) and its disease COVID-19 have been confirmed worldwide, with more than half a million deaths [1]. Spain has been one of the most affected countries. Previous studies on viral respiratory infections have shown pregnant women to be at higher risk of obstetric and perinatal complications due to changes in their immune response [2]. Reports from the beginning of the pandemic suggest that pregnant women are at an increased risk of developing the more severe disease compared to the general population but also may suffer increased adverse perinatal outcomes [3]. Where systematic screening has been performed at admission for delivery, approximately 14% of women were found to be asymptomatic SARS-CoV-2-positive [4,5]. An increased rate of prelabor rupture of membranes in pregnancies of women with SARS-CoV-2 has previously been reported in a case series of symptomatic infections, including our population [6,7,8]. The obstetric outcome of asymptomatic infected pregnancies has not been well documented.

The Spanish Obstetric Emergency group urgently changed its objectives on 8 March 2020 to focus on documenting SARS-CoV-2 infection during pregnancy. Screening was commenced in all pregnant women admitted for delivery and continues to date across Spain. We found that around 2% of mothers, with no suspected infection or symptoms, test positive for SARS-CoV-2 [9]. These mothers, families, and care providers face childbirth with uncertainty on a daily basis. As information is lacking on perinatal characteristics and birth outcomes regarding asymptomatic SARS-CoV-2 infection at term, evidence-based counseling for mothers is limited. The objective of this study was to determine if SARS-CoV-2 infection before delivery among asymptomatic mothers, compared to SARS-CoV-2 negative asymptomatic pregnancies, had different obstetric outcomes.

## 2. Materials and Methods 

This was a multicenter prospective study of consecutive cases of SARS-CoV-2 infection in a pregnancy cohort registered by the Spanish Obstetric Emergency group in 42 hospitals [9]. The registry’s objective updates were approved by the coordinating hospital’s Medical Ethics Committee on 23 March 2020 (reference number: PI 55/20); each collaborating center subsequently obtained protocol approval locally. The registry protocol is available at ClinicalTrials.gov, identifier NCT04558996. A complete list of authors and centers contributing to the study is provided as Supplementary Table S1. Upon recruitment, given the contagiousness of the disease and the lack of personal protection equipment, mothers consented by either signing a document, when possible, or verbally, which was recorded in the patient’s chart. A specific database was designed for recording information regarding SARS-CoV-2 infection in pregnancy. Data were entered by the lead researcher for each center after delivery, with a follow-up of six weeks postpartum in order to detect complications or symptomatic infections. We developed an analysis plan using the recommended contemporaneous methods and followed existing STROBE guidelines for reporting our results (Supplementary Table S2) [10]. 

**Figure 1:**
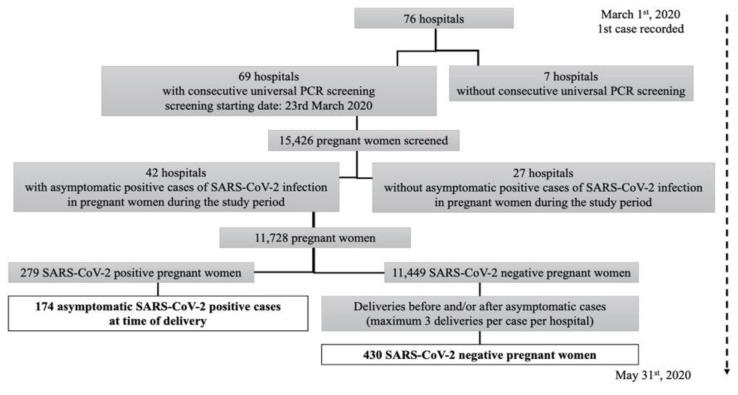
Flow chart of the study data. PCR, polymerase chain reaction; SARS-CoV-2, severe acute respiratory syndrome coronavirus 2.

We included all asymptomatic obstetric patients, detected by screening for SARS-CoV-2 infection at admission to the delivery ward during the study period (23 March to 31 May 2020). We excluded women with symptoms during the antenatal period, at delivery, or during the postpartum six-week follow-up. SARS-CoV-2 infection was diagnosed by positive double-sampling polymerase chain reaction (PCR) from nasopharyngeal swabs. Noninfected patients were those defined as a negative PCR at admission to delivery, and with no symptoms pre- or post-partum. Each center provided between two and three PCR negative asymptomatic pregnancies per asymptomatic infected mother, by providing either a standardized randomization table or by selecting negative asymptomatic pregnancies that delivered immediately before and after each asymptomatic infected mother (Figure 1). This method was deployed to adjust for center conditions and management at the time of delivery and to decrease the risk of selection bias. Follow-up was performed up to six weeks postpartum for all patients to verify that symptoms did not develop and to ascertain birth outcomes.

Information regarding the demographic characteristics of each pregnant woman, comorbidities, and previous and current obstetric history was extracted from the clinical and verbal history of the patient. For perinatal events, we recorded gestational age (GA) at delivery, preterm delivery (below 37 weeks), stillbirth, the onset of labor, type of delivery, prelabor rupture of membranes (PROM), preterm prelabor rupture of membranes (PPROM), gestational hypertension, preeclampsia, obstetric hemorrhage, and thrombotic risk. Neonatal data included sex, birth weight, one- and five-minute Apgar scores, umbilical artery pH, and neonatal intensive care unit (NICU) admission. Definitions of clinical and obstetric conditions followed international criteria [11,12,13]. 

The studied variables were tested for normal distribution using Kolmogorov–Smirnov or Shapiro–Wilk tests. Descriptive data are presented as median (range), or percentage (number). *p*-values were obtained by Mann–Whitney’s U test for numerical variables and Pearson chi-squared or Fisher’s exact test for categorical variables. A *p*-value below 0.05 was considered statistically significant. 

To compute the association of SARS-CoV-2 infection with obstetric outcomes of interest that were statistically significant in the univariate analysis, the potential influence of known and suspected measured confounding factors was controlled for with multivariable logistic regression modeling. We derived the adjusted odds ratios (aOR) with 95% confidence intervals (95% CI) after checking scientifically sound two-way interactions. The selection process for variables was driven by causal knowledge for the adjustment of confounders, verifying the statistical association of potential confounding factors with SARS-CoV-2 infection and the obstetric outcomes of interest (excluding intermediate variables of the causal chain), and it was based on previous findings and clinical constraints [11,12,13]. The complete list of covariates included in the maximum multivariable logistic regression model for the outcome of interest, after verifying the absence of significant interactions with SARS-CoV-2 infection, is as follows: multiple pregnancies, threatened abortion, ethnicity (categorized as Caucasian vs. non-Caucasian), smoking (categorized as current smokers/ex-smokers vs. non-smokers), chronic lung comorbidities (excluding asthma), and nulliparity, in accordance with the ten-to-one event per variable rule to avoid model overfitting [14]. Modeling was performed after excluding pregnancies with missing data; nulliparity had 1.4% missing values, whereas the other variables had none. A confounder remained in the model if the coefficient for SARS-CoV-2 infection changed by more than ten percent when the potential confounder was removed. Data were analyzed using the IBM SPSS Statistics 23 statistical package (Armonk, NY, USA: IBM Corp.); regression analyses were performed with the lme4 package in R, version 3·4 (RCore Team, 2017) [15].

## 3. Results

### 3.1. Main Results

#### 3.1.1. General Date

- During the study period, 11,728 patients were screened in 42 centers [9].
- A total of 279 (2.4%) SARS-CoV-2 positive patients were identified, of which 174 patients were asymptomatic at pregnancy, admission, and during postpartum follow-up (62% of the infected population).
- A total of 430 asymptomatic PCR negative patients were included.

#### 3.1.2. Baseline Characteristics

- No differences were found in anthropometric variables.
- The asymptomatic infection group showed a significantly higher proportion of Latin American (*p* = 0·002) and Black ethnicities (*p* = 0·003) compared to the noninfected group.
- Parity and blood type showed no SIGNIFICANT differences.
- Maternal comorbidities evaluated were also similar between study groups (Table 1).
- Obstetric history showed no differences between groups.

#### 3.1.3. Pregnancy Characteristics

- There were no significant differences between the proportions of singleton pregnancies, pregnancies by in vitro fertilization, those at high-risk for preeclampsia, thrombotic risk, or prophylactic treatment with either aspirin or heparin, the incidence of fetal anomalies, short cervix, vaccination, threatened preterm labor, or onset of labor.
- In total, 62 (35.6%) asymptomatic patients were hospitalized antenatally, compared to 49 (11.4%) noninfected patients (*p* < 0.001).
- Finally, there were no differences in the requirement of an intensive care unit for the mother, although one case from the asymptomatic group required intubation for noninfection-related complications (i.e., general anesthesia complications in a cesarean section due to placental abruption) (Table 2).

#### 3.1.4. Obstetric Outcomes

- No differences between GA at delivery, the incidence of preterm delivery (7.5% infected vs. 6.5% noninfected, OR = 1.16, 95% CI 0.59–2.29; *not significant (NS)*), cesarean section (20.7% infected vs. 17.2% noninfected, OR 1.25, 95% CI 0.81–1.95; *NS*) or stillbirth between groups.
- OR of PROM at term (≥37 weeks of gestation) were notably higher in the infected group (17.8% infected vs. 10.2% noninfected, OR 1.90, 95% CI 1.16–3.13; *p* = 0.011), as well as when only nulliparous women were analyzed (26.2% infected vs. 11.9% noninfected, OR 2.63, 95% CI 1.29–5.36, *p* = 0.007).
- Significant differences were found neither in the mode of delivery, gestational hypertension, preeclampsia, or obstetric hemorrhage, nor in PPROM (38.5% infected vs. 17.9% noninfected, OR 2.88, 95% CI 0.69–12.05; *NS*).
- Finally, no maternal deaths were reported in both groups (Table 3).

#### 3.1.5. Neonatal Data

- Neonatal variables also showed no differences in sex, birth weight, Apgar scores, or umbilical artery pH
- There was a significant difference between admission to the NICU between groups (6.9% infected vs. 1.6% noninfected, OR 4.48, 95% CI 1.73–11.55; *p* = 0.001), although the length of hospitalization was similar (median 13.2 days infected vs. 11.2 days noninfected; *NS*) (Table 3).

#### 3.1.6. Multivariable Logistic Model 

The final estimated multivariable logistic model, after excluding eight pregnancies with missing data, confirmed the association of SARS-CoV-2 infection with PROM among term pregnancies. An 88% increase in PROM occurred in the asymptomatic SARS-CoV-2 positive group compared to the negative group (aOR 1.88, 95% CI 1.13–3.11; *p* = 0.015), with no other covariate selected by the model (Table S3).

## 4. Discussion

To our knowledge, based on a thorough inspection of the obstetric COVID-19 literature, this is the first study to compare totally asymptomatic SARS-CoV-2 positive mothers with those not infected at delivery. Our results show a significant difference in the odds of PROM among pregnancies at term in a multivariate model, a feature not previously described.

Approximately 60% of SARS-CoV-2 positive patients identified at delivery were asymptomatic, which agrees with previously published data in our setting [4]. Baseline characteristics of our participants show a higher proportion of Latin American and Black ethnicity in the asymptomatic infected patients, which is consistent with previous literature suggesting a higher risk of infection, and, therefore, obstetric findings were adjusted for this characteristic. This higher risk of infection may be explained by socioeconomic factors, such as the type of work performed, family cohabitation, and comorbidities associated with ethnicity [16]. We found no differences in blood group type, comorbidities, or obstetric history, which have been reported as associated with progression and aggression of the infection [17,18].

Hospitalization before delivery was higher in infected mothers, a feature that is likely to be associated with the diagnosis of SARS-CoV-2 infection; in the early stages of the pandemic, many centers were not prepared to discharge a woman home upon confirming a SARS-CoV-2 infection. Many of these patients stayed for observation and isolation, with an average hospitalization stay of one day. On the other hand, maternal intensive care unit requirements were not different between groups, and less than those reported for symptomatic infections, as expected [19].

Our main finding is a higher proportion of PROM among term pregnancies (beyond 37 weeks of gestation) in the asymptomatic cohort when compared to noninfected women. This proportion was particularly important in the group of nulliparous women, which have no previous pregnancy risk factors that may increase PROM, such as cesarean section, previous preterm delivery, or adverse perinatal outcomes such as preeclampsia. The association of asymptomatic infection by SARS-CoV-2 with PROM has not been previously reported, mainly because at the beginning of the pandemic, universal screening was not performed upon admission to delivery wards, and secondly, because there are no previous reports comparing asymptomatic infected and noninfected patients. A previous screening study showed that approximately 14% of women were asymptomatic SARS-CoV-2-positive patients when admitted for delivery [5]; this has been recently confirmed by antibody testing from maternal serum in the first and third trimesters of pregnancy in our setting [4], which suggests that initial studies reporting on SARS-CoV-2 infection in pregnant women probably presented a selection bias regarding the presence of symptoms.

Asymptomatic infection of SARS-CoV-2 may be associated with abnormal imaging, with studies suggesting subclinical repercussion and altered immunity [20]. There are reports suggesting that a considerable number of asymptomatic patients may have lung opacities on computer tomography, with ground-glass opacities and consolidation [21]. Other findings reported in asymptomatic infections are lymphopenia, thrombocytopenia, elevated liver enzymes, and a decreased immune response [20]. Subclinical infections in pregnancy may be associated with PROM by various mechanisms, such as activation of inflammation. However, this has usually been associated with symptomatic infections [22].

Our findings support the hypothesis that a subclinical SARS-CoV-2 infection in pregnancy may result in PROM. However, it does not appear to be associated with increased preterm delivery or neonatal risks, probably because the asymptomatic status indicates a lower level of infective ailment. In the case of PPROM, the observed odds were consistent with PROM results, but potentially due to a lack of power, the possible association of SARS-CoV-2 infection with this outcome could not be confirmed. Perinatal outcomes were similar between our groups and more favorable than those reported in symptomatic patients, with lower rates of caesarean section, prematurity, thrombotic disease, preeclampsia, and growth restriction [3,19]. This leads us to believe that many obstetric outcomes may be related to maternal symptom severity. In the absence of symptoms, mothers can be reassured about perinatal outcomes. Likewise, the observed high NICU admission rate may be influenced by the presence of the infection, as many neonates may have been admitted for observation due to the maternal diagnosis. There have recently been arguments for and against universal screening in labor wards because of stigmatization and risk of separation of the mother and neonate [23]. We believe that our results from asymptomatic patients favor universal screening for protection of healthcare workers, warning clinicians that further protective measures should be undertaken, but symptoms and maternal clinical severity may be the determinant factors with regard to the management of the neonate and the possibility of direct breastfeeding without mother–baby separation, with adequate hygienic measures [24,25].

Our study has various strengths: in particular, a prospective screening program with a well-designed database which allowed us to record many characteristics of this novel infection. We evaluated outcomes during the antenatal period, at delivery, and during the postpartum six-week follow-up, in a uniform way for both the infected cohort and the uninfected comparison group. The SARS-CoV-2 negative comparison group was selected from the same centers where the infected mothers delivered and within the same timeframe so as to have similar conditions, thereby minimizing selection and performance biases. Both groups were well defined: more than one-third of the SARS-CoV-2 positive patients were antenatally admitted for observation, and every study participant follow-up was completed 6-week postpartum, allowing us to exclude mothers who became symptomatic after birth. We are therefore confident about establishing the asymptomatic status of mothers in the study. Multivariable analyses allowed for control of confounding variables included in the model. We acknowledge as a limitation the absence of the complete screened cohort; thus, our study has a hybrid design comprised of a prospective cohort of cases, with controls delivered immediately before and after each asymptomatic infected mother, representative of our population. The PCR negative comparison group was a subsample of the screen-negative cohort from all 42 hospitals that had PCR positive asymptomatic mothers. However, the concurrent method applied for the selection of a noninfected cohort allowed for a comparison unaffected by the difference in time of exposure and outcome assessment. We believe our findings are trustworthy, and the multicenter nature of the study adds to its generalizability.

## 5. Conclusions

Totally asymptomatic women with SARS-CoV-2 infection in pregnancy had higher PROM among term pregnancies compared to noninfected patients. Neonates had higher NICU admission rates, likely due to the isolation and observation protocols associated with maternal PCR positive status. We believe screening for SARS-CoV-2 infection in pregnant patients at admission to delivery may be necessary for the protection of healthcare workers. However, the presence of symptoms and maternal clinical severity appear to be the most relevant factors to determine mother–baby separation and direct breastfeeding, with adequate hygiene measures. Therefore, pregnant women who are SARS-CoV-2 positive at admission to the delivery ward should be reassured by their healthcare workers in the absence of symptoms, as low risk of infection-related obstetric morbidity and adverse outcomes are observed.

## Figures and Tables

**Table 1:** Baseline characteristics of the study participants (*n* = 604).

Variable	Asymptomatic SARS-CoV-2 Positive Patients *n* = 174	SARS-CoV-2 Negative Patients *n* = 430	*p*-Value
**Maternal characteristics**			
Maternal age (years)	32.6 (18–45)	33.2 (19–49)	*NS*
Maternal height (cm)	163.3 (150–179)	163.4 (150–180)	*NS*
Maternal weight (Kg)	71.0 (42–123)	70.6 (43–117)	*NS*
Maternal body mass index (Kg/m^2^)	26·6 (16.0–48.1)	26.5 (16·9–44.5)	*NS*
Obesity	22 (12.6)	63 (14.7)	*NS*
**Ethnicity**			
Caucasian	115 (66.1)	348 (80.9)	<0.001
Latin American	34 (19.5)	44 (10.2)	0.002
Black	8 (4.6)	3 (0.7)	0.003
Other	17 (9.8)	35 (8.2)	*NS*
Nulliparity	65/171 (38.0)	177/425 (41.6)	*NS*
A blood type	67/153 (43.8)	157/336 (46.7)	*NS*
Positive Rh status	134/153 (87.6)	300/336 (89.3)	*NS*
**Maternal comorbidities**			
Chronic cardiac disease	1 (0.6)	3 (0.7)	*NS*
Chronic lung disease	1 (0.6)	1 (0.2)	*NS*
Asthma	2 (1.1)	12 (2.8)	*NS*
Thrombophilia	2 (1.1)	6 (1.4)	*NS*
Anemia	5 (2.9)	22 (5.1)	*NS*
Pregestational diabetes with insulin treatment	0 (0.0)	0 (0.0)	
Smoking	21 (12.1)	55 (12.8)	*NS*
Pregestational hypertension	2 (1.1)	2 (0.5)	*NS*
**Obstetric history**			
Previous gestational hypertension	5 (2.9)	7 (1.6)	*NS*
Previous gestational thrombocytopenia	3 (1.7)	2 (0.5)	*NS*
Previous severe preeclampsia	3 (1.7)	2 (0.5)	*NS*
Previous growth restriction	9 (5.2)	14 (3.3)	*NS*

Data shown as median(range), or *n* (percentage). *p*-values obtained by Mann–Whitney’s U test for numerical variables and Pearson chi-squared or Fisher’s exact test for categorical variables. SARS-CoV-2, severe acute respiratory syndrome coronavirus 2; *NS*, nonsignificant.

**Table 2:** Pregnancy characteristics of the study participants (*n* = 604).

Variable	Asymptomatic SARS-CoV-2 Positive Patients *n* = 174	SARS-CoV-2 Negative Patients *n* = 430	*p*-Value
**Current pregnancy characteristics**			
Singleton pregnancy	170 (97.7)	418 (97.2)	*NS*
In vitro fertilization	11 (6.3)	14 (3.3)	*NS*
Threatened abortion	9 (5.2)	10 (2.3)	*NS*
High-risk for preeclampsia in 1rst trimester	6 (3.4)	24 (5.6)	*NS*
Aspirin prophylaxis	14 (8.0)	23 (5.3)	*NS*
Low-molecular weight heparin prophylaxis	8 (4.6)	11 (2.6)	*NS*
Fetal anomaly in first trimester	1 (0.6)	2 (0·5)	*NS*
Fetal anomaly in anatomy scan	1 (0.6)	8 (1.9)	*NS*
Group B Streptococcus infection	25 (14.4)	57 (13.3)	*NS*
Clinical and ultrasound prematurity screening	3/162 (1.9)	13/353 (3.7)	*NS*
Influenza vaccination	85/156 (54.5)	164/352 (46.6)	*NS*
Pertussis vaccination	146/163 (89.6)	342/377 (90.7)	*NS*
Threatened preterm labor	4 (2.3)	14/384 (3.6)	*NS*
Hospitalization before labor	62 (35.6)	49 (11.4)	<0·001
**Onset of labor**			
Elective caesarean section	12 (6.9)	19 (4.4)	*NS*
Spontaneous	94 (54.0)	264 (61.4)	
Induced	68 (39.1)	147 (34.2)	
**Thrombotic risk**			*NS*
Low	143 (82.2)	328 (76.3)	*NS*
Medium	28 (16.1)	85 (19.8)	*NS*
High	3 (1.7)	13 (30)	*NS*
Intensive care unit required	1 (0.6)	2 (0.5)	*NS*
Intubation required	1 (0.6)	0 (0.0)	*NS*

Data shown as *n* (percentage). *p*-values obtained by Pearson chi-squared or Fisher’s exact test for categorical variables. SARS-CoV-2, severe acute respiratory syndrome coronavirus 2; *NS*, nonsignificant.

**Table 3:** Perinatal and neonatal data of the study population (*n* = 604).

Variable	Asymptomatic SARS-CoV-2 Positive Patients *n* = 174	SARS-CoV-2 Negative Patients *n* = 430	*p*-Value
**Perinatal data**			
Gestational age at delivery (weeks)	39.0 (31–42)	39.1 (28–42)	*NS*
Preterm delivery (below 37 weeks)	7.5 (13)	6.5 (28)	*NS*
Stillbirth	1.1 (2)	0.0 (0)	*NS*
PROM	17.8 (31)	10.2 (44)	0.011
PROM in nulliparous women	26.2 (17/65)	11.9 (21/177)	0.007
PPROM	2.9 (5)	1.2 (5)	*NS*
**Onset of labor**			
Programmed caesarean section	6.9 (12)	4.4 (19)	*NS*
Spontaneous	54.0 (94)	61.4 (264)	
Induced	39.1 (68)	34.2 (147)	
**Type of delivery**			
Caesarean	20.7 (36)	17.2 (74)	*NS*
Vaginal	70.1 (122)	67.2 (289)	
Vacuum or forceps	9.2 (16)	15.6 (67)	
Gestational hypertension	6.4 (11)	5.8 (24)	*NS*
**Preeclampsia**			
Mild/moderate	2.3 (4)	4.9 (21)	*NS*
Severe	1.7 (3)	0.5 (2)	
Obstetric hemorrhage	3.4 (6)	4.7 (20)	*NS*
**Thrombotic risk**			
Low	82.2 (143)	76.3 (328)	*NS*
Medium	16.1 (28)	19.8 (85)	
High	1.7 (3)	3.0 (13)	
**Neonatal data**			
Male sex	50.6 (86/170)	48.2 (205/425)	*NS*
Birth weight (grams)	3187 (315–4640)	3249 (900–4610)	*NS*
1’ Apgar score	9 (0–10)	9 (1–10)	*NS*
5’ Apgar score	10 (0–10)	10 (5–10)1	*NS*
Umbilical artery pH	7.26 (7.04–7.42)	7.26(6.90–7.46)	*NS*
NICU admission	6.9 (12)	1.6 (7)	0.001
Days in NICU	13.2 (1–48)	11.2 (5–17)	*NS*
**Cause of NICU admission:**			*NS*
Prematurity	25 (3/12)	71.4 (5/7)	*NS*
Respiratory distress	16.7 (2/12)	14.3 (1/7)	*NS*
COVID-19 protocol	33.3 (4/12)	0.0 (0/7)	*NS*

Data are shown as mean/range, or percentage(*n*). *p*-values obtained by Mann–Whitney’s U test for continuous variables and Pearson chi-squared or Fisher’s exact test for categorical variables. SARS-CoV-2, severe acute respiratory syndrome coronavirus 2; *NS*, nonsignificant; PROM, Premature rupture of membranes; PPROM, Preterm premature rupture of membranes; NICU, neonatal intensive care unit.

## Data Availability

The data presented in this study are available on request from the corresponding author. The data are not publicly available due to the multicenter nature of the study.

## Supplementary Materials

The following are available online at https://www.mdpi.com/1999-4915/13/1/112/s1, Table S1: List of authors and hospital members of the Spanish Obstetric Emergency Group included in this study (*n* = 42), Table S2: STROBE Statement—a checklist of items that should be included in reports of observational studies, Table S3: Odds ratio (OR) and adjusted odds ratio (aOR) with the corresponding 95% confidence intervals and *p*-values for the outcomes associated with SARS-CoV-2 infection in pregnancy.
