# Supplementary material for: Obstetric Outcomes of SARS-CoV-2 Infection in Asymptomatic Pregnant Women
Source: Viruses. 2021 Jan 15;13(1):112. doi: 10.3390/v13010112 (PMC7830626; doi:10.3390/v13010112)
Supplement: Supplementary file 1 [file viruses-13-00112-s001.pdf]

viruses

MDPI

*Supplementary Materials*

# Obstetric outcomes of SARS-CoV-2 infection in asymptomatic pregnant women.

**Table S1.** List of authors and hospitals members of the Spanish Obstetric Emergency Group included in this study (*n* = 42).

| Hospital | Principal Investigator | e-mail |
|---|---|---|
| Santa Creu i Sant Pau University Hospital | Mónica Cruz Lemini | mcruzl@santpau.cat |
| Parc de Salut Mar University Hospital | Elena Ferriols Pérez | eferriols@psmar.cat |
| Fundación de Investigación Biomédica, Hospital Universitario Puerta de Hierro | María Luisa de la Cruz Conty | farmcruz@gmail.com |
| Hospital Universitario San Cecilio, Instituto de Investigación Biosanitaria de Granada | África Caño Aguilar | africacano59@gmail.com |
| Hospital Universitario Puerta de Hierro | María Begoña Encinas Pardilla | beenpar@yahoo.es |
| Hospital Universitari Dexeus - Grupo Quirónsalud | Pilar Prats Rodríguez | pilpra@dexeus.com |
| Hospital Universitario La Paz | Marta Muner Hernando | mlm_marta@hotmail.com |
| Hospital Doce de Octubre | Laura Forcén Acebal | lauratrona@gmail.com |
| Hospital General Universitario Gregorio Marañón | Pilar Pintado Recarte | ppintadorec@yahoo.es |
| Hospital de Santa Creu i Sant Pau | María del Carmen Medina Mallén | mmedinam@santpau.cat |
| Hospital Clínico San Carlos | Noelia Pérez Pérez | perezpereznoelia@yahoo.es |
| Hospital de Santa Caterina | Judit Canet Rodriguez | judit.canet@ias.cat |
| Hospital Universitario de Salamanca | Ana Villalba Yarza | avillalba@saludcastillayleon.es |
| Hospital Universitario Quirónsalud Pozuelo de Alarcón | Olga Nieto Velasco | onietovela@hotmail.com |
| Hospital Univesitario de Getafe | Pablo Guillermo Del Barrio Fernández | pablobarri@gmail.com |
| Hospital Univesitario Severo Ochoa | Carmen María Orizales Lago | carmen.orizales@salud.madrid.org |
| Hospital La Fe | Beatriz Marcos Puig | beatrizmarcospuig@gmail.com |
| Hospital Sant Joan de Reus | Begoña Muñoz Abellana | begomunoz@hotmail.com |
| Hospital Universitario de Ferrol | Laura Fuentes Ricoy | laura.fuentes.ricoy@sergas.es |
| Hospital Universitario de Girona Doctor Josep Trueta | Águeda Rodríguez Vicente | aguedarv@gmail.com |
| Complejo Hospitalario A Coruña | María Jesús Janeiro Freire | mjaneirof@yahoo.es |
| HM Hospitales | Macarena Alférez Álvarez-Mallo | macarena.alferez.am@gmail.com |
| Hospital de Torrejón | Cristina Casanova Pedraz | casanovapedraz@hotmail.com |
| Hospital d'Inca | Onofre Alomar Mateu | alomarmateu@gmail.com |
| Hospital Parc Taulí | Cristina Lesmes Heredia | clesmes@tauli.cat |
| Hospital San Pedro Alcántara | Juan Carlos Wizner de Alva | jcwizner@gmail.com |
| Hospital Universitario Río Hortega | Alma Posadas San Juan | almapsj@gmail.com |
| Hospital Arnau de Vilanova | Montse Macià Badia | mmaciabadia@gmail.com |
| Hospital Clínico Universitario de Valladolid | Cristina Álvarez Colomo | calvarezc.cac@gmail.com |

| | | |
|---|---|---|
| Hospital General Universitario de Ciudad Real | Antonio Sanchez Muñoz | asanchezm@sescam.jccm.es |
| Hospital Universitari Germans Trias i Pujol | Laia Pratcorona Alicart | lpratcorona.mn.ics@gencat.cat |
| Hospital Universitario de Burgos | Rubén Alonso Saiz | alonsorub@yahoo.es |
| Hospital Universitario de Tarragona Juan XXIII | Mónica López Rodríguez | monicalopez4099@gmail.com |
| Hospital Universitario Infanta Sofía | María del Carmen Barbancho López | mbarbancholopez@gmail.com |
| Hospital de Poniente | Marta Ruth Meca Casbas | martameca@hotmail.com |
| Hospital Universitario de Cabueñes | Óscar Vaquerizo Ruiz | oscar.vaquerizo@sespa.es |
| Hospital Universitario Son Espases | Eva Morán Antolín | emoranantolin@yahoo.es |
| Hospital Virgen de la Luz | María José Núñez Valera | m.jose.nunez.valera@gmail.com |
| Complejo Asistencial de León | Camino Fernández Fernández | caminoffernandez@gmail.com |
| Hospital Universitario Son Llàtzer | Albert Tubau Navarra | atubau68@icloud.com |
| Hospital del Tajo | Alejandra Maria Cano Garcia | sandracano80@hotmail.com |
| Hospital Jerez de la Frontera | Susana Soldevilla Pérez | soldevillasusana@hotmail.com |
| Hospital Universitario Araba-Txagorritxu | Irene Gastaca Abásolo | igastaca@gmail.com |
| Hospital Universitario Central de Asturias | José Adánez García | adanezjose@gmail.com |
| Hospital Universitario Virgen de las Nieves | Alberto Puertas Prieto | apuertas51@hotmail.com |
| Hospital Universitario Virgen de Valme | Rosa Ostos Serna | rosam.ostos.sspa@juntadeandalucia.es |
| Hospital Universitario Virgen Macarena | María del Pilar Guadix Martín | pilarguadix@gmail.com |
| Hospital Virgen de la Concha | Mónica Catalina Coello | mccoello@gmail.com |
| Parc de Salut Mar University Hospital | Silvia Espuelas Malón | sespuelas@parcdesalutmar.cat |
| G. Chacon (Viamed Santa Angela de la Cruz Hospital) | José Antonio Sainz Bueno | jsainz@us.es |
| Hospital Universitario Juan Ramón Jiménez | María Reyes Granell Escobar | mrgranell@gmail.com |
| Hospital Universitario Puerta de Hierro | Sara Cruz Melguizo | saracruz.gine@yahoo.es |
| Hospital Universitario Puerta de Hierro | Oscar Martinez Perez | oscarmartinezgine@gmail.com |

**Table S2.** STROBE Statement—checklist of items that should be included in reports of observational studies.

| | Item | Recommendation | Page |
|---|---|---|---|
| **Title and abstract** | 1 | (*a*) Indicate the study's design with a commonly used term in the title or the abstract | 1 and 4 |
| | | (*b*) Provide in the abstract an informative and balanced summary of what was done and what was found | 4 |
| **Introduction** | | | |
| Background/rationale | 2 | Explain the scientific background and rationale for the investigation being reported | 6 |
| Objectives | 3 | State specific objectives, including any prespecified hypotheses | 6 |
| **Methods** | | | |
| Study design | 4 | Present key elements of study design early in the paper | 6-9 |
| Setting | 5 | Describe the setting, locations, and relevant dates, including periods of recruitment, exposure, follow-up, and data collection | 6-9, Figure 1 and Table S1 |
| Participants | 6 | (*a*) *Cohort study*—Give the eligibility criteria, and the sources and methods of selection of participants. Describe methods of follow-up<br>*Case-control study*—Give the eligibility criteria, and the sources and methods of case ascertainment and control selection. Give the rationale for the choice of cases and controls<br>*Cross-sectional study*—Give the eligibility criteria, and the sources and methods of selection of participants | 6-7 and Figure 1 |
| | | (*b*) *Cohort study*—For matched studies, give matching criteria and number of exposed and unexposed<br>*Case-control study*—For matched studies, give matching criteria and the number of controls per case | 7 and Figure 1 |
| Variables | 7 | Clearly define all outcomes, exposures, predictors, potential confounders, and effect modifiers. Give diagnostic criteria, if applicable | 7-9 and registry protocol: ClinicalTrials.gov, NCT04558996 |
| Data sources/ measurement | 8* | For each variable of interest, give sources of data and details of methods of assessment (measurement). Describe comparability of assessment methods if there is more than one group | 8 and registry protocol: ClinicalTrials.gov, NCT04558996 |
| Bias | 9 | Describe any efforts to address potential sources of bias | 7-9 |
| Study size | 10 | Explain how the study size was arrived at | 7 and Figure 1 |
| Quantitative variables | 11 | Explain how quantitative variables were handled in the analyses. If applicable, describe which groupings were chosen and why | 8 and registry protocol: ClinicalTrials.gov, NCT04558996 |
| Statistical methods | 12 | (*a*) Describe all statistical methods, including those used to control for confounding | 8-9 and registry protocol: ClinicalTrials.gov, NCT04558996 |
| | | (*b*) Describe any methods used to examine subgroups and interactions | 8-9 |
| | | (*c*) Explain how missing data were addressed | 8-9 |
| | | (*d*) *Cohort study*—If applicable, explain how loss to follow-up was addressed<br>*Case-control study*—If applicable, explain how matching of cases and controls was addressed<br>*Cross-sectional study*—If applicable, describe analytical methods taking account of sampling strategy | Registry protocol: ClinicalTrials.gov, NCT04558996 |
| | | (*e*) Describe any sensitivity analyses | No sensitivity analysis was carried out |

| | | | |
|---|---|---|---|
| **Results** | | | |
| Participants | 13* | (a) Report numbers of individuals at each stage of study—eg numbers potentially eligible, examined for eligibility, confirmed eligible, included in the study, completing follow-up, and analysed | Figure 1 |
| | | (b) Give reasons for non-participation at each stage | 7 and Figure 1 |
| | | (c) Consider use of a flow diagram | Figure 1 |
| Descriptive data | 14* | (a) Give characteristics of study participants (eg demographic, clinical, social) and information on exposures and potential confounders | 9-10 and Tables 1-2 |
| | | (b) Indicate number of participants with missing data for each variable of interest | Tables 1-3 |
| | | (c) *Cohort study*—Summarise follow-up time (eg, average and total amount) | 7-8 |
| Outcome data | 15* | *Cohort study*—Report numbers of outcome events or summary measures over time | 10 and Table 3 |
| | | *Case-control study*—Report numbers in each exposure category, or summary measures of exposure | |
| | | *Cross-sectional study*—Report numbers of outcome events or summary measures | |
| Main results | 16 | (*a*) Give unadjusted estimates and, if applicable, confounder-adjusted estimates and their precision (eg, 95% confidence interval). Make clear which confounders were adjusted for and why they were included | 10 (and 9) |
| | | (*b*) Report category boundaries when continuous variables were categorized | No continuous variables were categorized |
| | | (*c*) If relevant, consider translating estimates of relative risk into absolute risk for a meaningful time period | |
| Other analyses | 17 | Report other analyses done—e.g. analyses of subgroups and interactions, and sensitivity analyses | 9-10 and Table 3 |
| Key results | 18 | Summarise key results with reference to study objectives | 10-11 |
| Limitations | 19 | Discuss limitations of the study, taking into account sources of potential bias or imprecision. Discuss both direction and magnitude of any potential bias | 11 |
| Interpretation | 20 | Give a cautious overall interpretation of results considering objectives, limitations, multiplicity of analyses, results from similar studies, and other relevant evidence | 11-13 |
| Generalisability | 21 | Discuss the generalisability (external validity) of the study results | 11 |
| **Other information** | | | |
| Funding | 22 | Give the source of funding and the role of the funders for the present study and, if applicable, for the original study on which the present article is based | 14 |

*Give information separately for cases and controls in case-control studies and, if applicable, for exposed and unexposed groups in cohort and cross-sectional studies.

**Note:** An Explanation and Elaboration article discusses each checklist item and gives methodological background and published examples of transparent reporting. The STROBE checklist is best used in conjunction with this article (freely available on the Web sites of PLoS Medicine at http://www.plosmedicine.org/, Annals of Internal Medicine at http://www.annals.org/, and Epidemiology at http://www.epidem.com/). Information on the STROBE Initiative is available at www.strobe-statement.org.

**Table S3.** Odds ratio (OR) and adjusted odds ratio (aOR) with the corresponding 95% confidence intervals and p-values for the outcomes associated with SARS-CoV-2 infection in pregnancy.

| | | **Outcomes** | | | |
|---|---|---|---|---|---|
| | | **Hospitalization before labor** | **PROM*** | **PROM in nulliparous women** | **NICU admission** |
| **Univariate analysis (OR)** | | | | | |
| SARS-CoV-2 positive | | 4.30 | 1.90 | 2.63 | 4.48 |
| | 95% CI | 2.80 – 6.61 | 1.16 – 3.13 | 1.29 – 5.36 | 1.73 – 11.55 |
| | *p* | <0.001 | 0.011 | 0.007 | 0.001 |
| **Multivariate analysis (aOR)** | | | | | |
| SARS-CoV-2 positive | | | 1.88 | | |
| | 95% CI | | 1.13 – 3.11 | | |
| | *p* | | 0.013 | | |
| Multiple pregnancy | | | -- | | |
| | 95% CI | | -- | | |
| | *p* | | *NS* | | |
| Threatened abortion | | | -- | | |
| | 95% CI | | -- | | |
| | *p* | | *NS* | | |
| Ethnicity | | | -- | | |
| | 95% CI | | -- | | |
| | *p* | | *NS* | | |
| Smoking | | | -- | | |
| | 95% CI | | -- | | |
| | *p* | | *NS* | | |
| Chronic lung comorbidities | | | -- | | |
| | 95% CI | | -- | | |
| | *p* | | *NS* | | |
| Nulliparity | | | -- | | |
| | 95% CI | | -- | | |
| | *p* | | *NS* | | |

PROM, Premature Rupture of Membranes at term; NICU, Neonatal Intensive Care Unit.
*Multivariable logistic regression used for PROM as dependent variable and SARS-CoV-2 infection in pregnancy and known/suspected confounding variables as independent variables (see Materials and Methods for details).
-- Variables not held in the multivariate model

The selection process for covariates included in the maximum multivariable logistic regression model for PROM was as follows: by univariate analyses, the statistical association of both potential confounding factors with SARS-CoV-2 infection and potential confounding factors with PROM, was tested. Those statistically significant were included as covariates in the multivariable model. However, there were other potential confounding factors that were not statistically associated with SARS-CoV-2 and/or with PROM in the previous analyses but were included in the multivariable model anyway, because they had previously been described as risk factors for PROM by the scientific literature and could confound the association of SARS-CoV-2

with the outcome of interest. This is the case of smoking [American College of O, Gynecologists' Committee on Practice B-O: Prelabor Rupture of Membranes: ACOG Practice Bulletin, Number 217. Obstet Gynecol 2020, 135(3):e80-e97] and other covariates. Once the maximum multivariable logistic regression model was constructed, and in order to achieve the final estimated model: a confounder remained in the model if the coefficient for SARS-CoV-2 infection changed more than ten percent when the potential confounder was removed.
